# Application of Bamboo Shoot Superfine Powder/κ-Carrageenan Composite Gel in Bread: Texture, Properties and Flavor

**DOI:** 10.3390/foods15183193

**Published:** 2026-09-09

**Authors:** Kailin Li, Jiakai Xu, Shuchun Xu, Xinyue Xue, Zhirui Wu, Baodong Zheng, Xianliang Luo

**Affiliations:** 1 Engineering Research Center of Fujian-Taiwan Special Marine Food Processing and Nutrition, Ministry of Education, Fuzhou 350002, China; 2 College of Food Science, Fujian Agriculture and Forestry University, Fuzhou 350002, China; 3 Fujian Lianhua Qigong Food Co., Ltd., Nanping 353000, China

**Keywords:** bamboo shoot shell superfine powder, κ-carrageenan, composite gel, bread quality, flavor regulation

## Abstract

Bread texture and flavor are largely governed by the development of its internal microstructure and the migration dynamics of water and volatile compounds. In this study, a bamboo shoot shell superfine powder/κ-carrageenan composite gel system (KCS) was developed, and the effects of different addition levels (0%, 5%, 10%, 15%, 20%) of KCS on bread performance, structure, and flavor were investigated. Compared with κ-carrageenan (KC), KCS formed a denser and more continuous composite network structure with higher elasticity, freeze–thaw stability, and thermal stability. Bread with KCS showed improved color, reduced hardness, and chewiness. Electronic nose and tongue indicated that KCS effectively suppressed the release of undesirable volatiles and taste-active substances. The addition of 10% KCS promoted the formation of a uniform fine honeycomb gluten network structure, inhibited starch retrogradation, and improved the thermal stability of bread. During storage (1–5 d), KCS reduced the water loss rate of bread, delayed the increase in hardness and chewiness, and maintained good springiness. Metabolomic analysis showed that KCS increased the precursors of small peptides involved in the Maillard reaction, thereby enriching flavor. KCS can effectively improve bread quality, enrich bread flavor, and delay aging, providing a new strategy for the high-value utilization of bamboo shoot processing by-products.

## 1. Introduction

Bamboo is a perennial plant of the subfamily Bambusoideae in the family Poaceae, which is rich in long fibers. There are approximately 1670 of bamboo species distributed across 22 countries worldwide. China is one of the countries with abundant bamboo resources, with a current bamboo forest area of 6.41 million hectares [1]. However, bamboo shoot processing generates substantial by-products, including shoot shells and bases, as only 40–50% of the biomass is utilized. Consequently, 17.64–22.05 million tons of bamboo shoot shells are discarded annually, with most treated as waste or low-value animal feed, resulting in significant resource loss and environmental burdens. Nevertheless, bamboo shoot shells contain bioactive components such as dietary fiber, polysaccharides, flavonoids, and sterols. Numerous studies have explored the extraction of dietary fiber from bamboo shoot by-products. Tian et al. [2] compared the effects of four extraction methods (enzymatic, ultrasound-assisted enzymatic, deep eutectic solvent, and ultrasound-assisted deep eutectic solvent) on soluble dietary fiber from bamboo shoots. To further improve dietary fiber yield from bamboo shoot shells, a combined strategy involving steam explosion, alkaline extraction, and microbial fermentation has been developed [3]. In addition, fermentation of bamboo shoot residues using the medicinal white-rot fungus *Inonotus obliquus* has been shown to modify the lignocellulosic architecture, thereby enhancing the yield, composition, and functional properties of dietary fiber [4]. Despite these advances, dietary fiber extraction generally requires additional processing steps and increases production costs. Direct utilization of superfine-ground bamboo shoot powder in suitable applications could therefore provide a cost-effective alternative while alleviating the accumulation of bamboo shoot by-products.

κ-carrageenan is a natural sulfated polysaccharide derived from red algae. In recent years, its application in functional food development has gained increasing attention. Carrageenan gels offer advantages such as a low required concentration, wide applicability, and high transparency [5]. Thus, they are widely used as fat replacers in carbohydrate-based matrices. However, κ-carrageenan gels suffer from high brittleness, poor mechanical properties, easy syneresis, and thermal decomposition at high temperatures. These issues can be addressed by preparing composite gels through synergistic interactions between κ-carrageenan and other food hydrocolloids [6]. The gel network of κ-carrageenan can also synergize with other dietary components, such as dietary fiber, protein, and polyphenols. In food gel systems, the introduction of dietary fiber not only helps retain its health benefits but also improves the gel structure and performance [7]. Studies have shown that insoluble dietary fiber can modulate gel structure and rheological properties through hydrogen-bonding or electrostatic interactions with proteins. For example, wheat bran fiber interacts with soy protein isolate (SPI) via hydrogen bonds, altering the water distribution in the system, promoting gelation, and enhancing gel strength, viscoelasticity, and water-holding capacity [7]. The incorporation of yeast dietary fiber was found to significantly influence the gelation behavior of konjac glucomannan/κ-carrageenan composite gels [8]. Specifically, yeast dietary fiber increased gel whiteness and hardness, while reducing chewiness, springiness, and gel strength. Wu et al. proposed using water-insoluble citrus fiber (ICF) to improve the stability and water-holding capacity of κ-carrageenan gels through network reinforcement [9]. Therefore, the complementary advantages of dietary fiber and κ-carrageenan may have a positive impact on the structure and performance of bread.

Superfine grinding technology is an advanced fine-crushing technique that can reduce materials to a micron scale and is an effective material modification technology [10]. Compared with conventional grinding, superfine grinding significantly alters the physicochemical properties of powders, such as oil-holding capacity, swelling power, and flowability, making the materials more suitable for production and processing needs [11]. In recent years, superfine grinding has been widely applied in the processing of fruits, vegetables, grains, legumes, and some Chinese medicinal herbs [12,13,14]. Superfine grinding can enhance the physicochemical properties of bamboo shoot dietary fiber and increase the release of bioactive compounds, enabling its application as a staple food improver, dietary fiber supplement, and functional food ingredient [15]. However, its potential use in gel-based systems and its effects on bread quality remain largely unexplored.

In this study, bamboo shoot shells were used as a raw material to prepare superfine powders via superfine grinding. A composite gel system of bamboo shoot superfine powder and κ-carrageenan was constructed, its structural characteristics were studied, and the effects of different addition levels of the composite gel on bread performance, structure, and flavor were further investigated. The results can provide new ideas for the utilization of bamboo shoot processing by-products and serve as a reference for improving bread quality and flavor characteristics.

## 2. Materials and Methods

### 2.1. Materials

Bamboo shoot shells (Lianhua Qigong Food Co., Ltd., Nanping, Fujian, China); κ-carrageenan (Xintai Industrial Co., Ltd., Shanghai, China); anhydrous ethanol (Sinopharm Chemical Reagent Co., Ltd., Shanghai, China); high-gluten wheat flour (Xinxiang Liangrun Whole Grain Food Co., Ltd., Xinxiang, Henan, China); yeast powder (Hubei Angel Yeast Co., Ltd., Yichang, Hubei, China); white sugar (Lianhua Holding Co., Ltd., Shanghai, China); butter (Yili Dairy Co., Ltd., Wuhan, China); and edible salt (Qinghai Salt Industry Co., Ltd., Xining, Qinghai, China).

### 2.2. Methods

#### 2.2.1. Determination of Basic Components of Bamboo Shoot Shells

According to the national standard methods of GB 5009.3-2016, GB 5009.4-2016, GB 5009.5-2016, GB 5009.6-2016, and GB 5009.88-2014 [16,17,18,19,20], the contents of moisture, ash, protein, fat, and dietary fiber in bamboo shoot shell samples were determined.

#### 2.2.2. Preparation of Bamboo Shoot Shell Superfine Powder

The bamboo shoot shells were cleaned, hot-air dried at 60 °C, and then coarsely ground using a high-speed pulverizer at 4200 r/min (DS-200, Changzhou Xiangtian Chemical Co., Ltd., Changzhou, Jiangsu, China). The ground material was passed through 40-mesh and 80-mesh sieves to obtain 40-mesh and 80-mesh coarse bamboo shoot shell powders, which were designated as 40-mesh and 80-mesh, respectively. The 40-mesh coarse powder (500 g per batch) was fed into a superfine grinder (HMB100B, Sanhe Yayuan Machinery Manufacturing Co., Ltd., Langfang, Hebei, China) and ground at 40 Hz for 15 min to obtain bamboo shoot shell superfine powder (S).

#### 2.2.3. Preparation of Bamboo Shoot Superfine Powder/κ-Carrageenan Composite Gel

κ-carrageenan, at concentrations of 0.7%, 0.7% and 1.2%, was dissolved in 60 °C hot water under stirring until completely dissolved. The bamboo shoot superfine powder, at the ratios of 2:1, 1:1, and 1:2 (0.35%, 0.7%, 2.4%), was added. After stirring for 20 min, the mixture was cooled to room temperature (25 °C) to obtain the composite gel matrices, denoted as KCS (2:1), KCS (1:1), and KCS (1:2). We set KC and S as the control samples. The preparation process of KC is as follows: κ-carrageenan at concentrations of 0.7% was dissolved in 60 °C hot water under stirring until completely dissolved. S represents bamboo shoot superfine powder.

#### 2.2.4. Preparation of Bread with Bamboo Shoot Superfine Powder/κ-Carrageenan Composite Gel

The bamboo shoot superfine powder/κ-carrageenan composite gel (KCS 1:1, in the non-solidified state), at addition levels of 0%, 5%, 10%, 15%, and 20%, was added to 100 g of high-gluten wheat flour (protein content was 13.2 g/100 g). After thorough mixing, 1 g of yeast powder, 10 g of white sugar, and 60 mL of warm water (30 °C) were added and mixed at a low speed in the dough mixer (HM770, Hauswirt Baking Appliances GmbH, Frankfurt, Germany) until no dry powder remained. Then, 7 g of butter and 0.6 g of edible salt were added, and mixing was switched to a high-speed until a smooth dough formed. The dough was divided into uniform small pieces (each approximately 30 g), proofed in a proofing cabinet at 37 °C for 45 min, and then baked for 10 min (top heat 180 °C, bottom heat 160 °C). The resulting breads were denoted as KCS-5, KCS-10, KCS-15, and KCS-20. KCS-0, KC, and S served as the control groups, representing white bread without composite gel, bread supplemented with 10% κ-carrageenan alone, and bread supplemented with 10% bamboo shoot superfine powder alone, respectively.

#### 2.2.5. Water-Holding Capacity (WHC) of Bamboo Shoot Shell Powder

The method of Zheng et al. [21] was modified. Briefly, 0.5 g samples (40-mesh, 80-mesh, S) were placed into a centrifuge tube, 20 mL of distilled water was added, and then shaken to mix thoroughly. Samples were then allowed to stand at room temperature for 24 h. After centrifugation (6000× *g*, 10 min), we weighed the tube. The water-holding capacity was calculated using the following formula:

(1) $$\begin{array}{c} WHC\;\left(g/g\right)\;=\;\frac{\left(m_{2}-m_{1}\right)}{m_{1}} \end{array}$$

where *m*_1_—the sample mass’s before water absorption, g; and *m*_2_—the sample’s mass after water absorption, g.

#### 2.2.6. Oil-Holding Capacity (OHC) of Bamboo Shoot Shell Powder

The method of Zheng et al. [21] was modified. Briefly, 0.5 g samples (40-mesh, 80-mesh, S) were placed into a centrifuge tube, 10 mL of corn oil was added, and then mixed thoroughly. Samples were then allowed to stand at room temperature for 2 h. After centrifugation (6000× *g*, 15 min), we weighed the tube. The oil-holding capacity was calculated using the following formula:

(2) $$\begin{array}{c} OHC\;\left(g/g\right)\;=\;\frac{\left(m_{2}-m_{1}\right)}{m_{1}} \end{array}$$

where *m*_1_—the sample’s mass before oil absorption, g; and *m*_2_—the sample’s mass after oil absorption, g.

#### 2.2.7. Water-Swelling Capacity (WSC) of Bamboo Shoot Shell Powder

The method of Zheng et al. [21] was modified. Briefly, 0.5 g samples (40-mesh, 80-mesh, S) were placed into a 10 mL graduated cylinder. The volume of the dry sample (*V*_1_) was read at eye level. Then, 8 mL of distilled water was added into the cylinder, mixed thoroughly, and gently stirred to remove air bubbles. After placing horizontally at room temperature for 24 h, we read the volume of the sample after swelling (*V*_2_). Each sample was measured three times, and the average value was taken. The water-swelling capacity was calculated using the following formula:

(3) $$\begin{array}{c} WSC\;\left(mL/g\right)\;=\;\frac{\left(V_{2}-V_{1}\right)}{W} \end{array}$$

where *V*_1_—the volume of dry sample, mL; *V*_2_—the volume of sample after swelling, mL; and *W*—the sample’s mass, g.

#### 2.2.8. Water-Holding Capacity (WHC) of Gels

Briefly, 5 g of gel samples were placed in a double-filter-paper centrifuge tube and allowed to stand at room temperature for 24 h. After centrifugation (3H24R1, Changsha Xiangzhi Centrifuge Instrument Co., Ltd., Changsha, Hunan, China) at 4000× *g* for 5 min, the sample weight was recorded (*m*_2_). The water-holding capacity was calculated using the following formula:

(4) $$\begin{array}{c} WHC\;\left(\%\right)=\frac{m_{2}}{m_{1}}\times 100 \end{array}$$

where *m*_1_—the sample’s mass before water loss, g; and *m*_2_—the sample’s mass after water loss, g.

#### 2.2.9. Particle Size Distribution Measurement of Bamboo Shoot Shell Superfine Powder

The particle size distribution of the samples was measured using a Malvern laser particle size analyzer (Mastersizer 3000, Malvern Instruments Ltd., Mrlvern, UK). Before measurement, the bamboo shoot superfine powder was uniformly dispersed in water. The refractive indices of the bamboo shoot superfine powder and pure water were set at 1.53 and 1.33, respectively. After measurement, the measuring cell and dispersion system were cleaned promptly. Each sample was measured three times, and the average particle size was recorded.

#### 2.2.10. Scanning Electron Microscopy (SEM) of Gels and Breads

The microstructure of freeze-dried samples was evaluated using scanning electron microscopy (Gemini SEM 300, Carl Zeiss AG, Oberkochen, Germany). The freeze-dried sample powder was applied onto conductive adhesive tape and sputter-coated with gold under a vacuum. Then, images of samples were recorded at magnifications of 500× [8].

#### 2.2.11. Fourier Transform Infrared Spectroscopy (FTIR) of Gels and Breads

Exactly 1 mg of sample powder was mixed with 100 mg of KBr (*w*/*w*) and ground under an infrared lamp. The mixture was pressed into a transparent disk using a hydraulic press (HY-12, TianGuang new optical instrument, Tianjin, China). And then the samples were examined using Fourier transform infrared spectrometer (Nicolet iS20, Thermo Fisher Scientific Inc., Waltham, MA, USA). Infrared spectra were recorded in the wavenumber range of 400–4000 cm^−1^, with 32 scans at a spectral resolution of 4 cm^−1^.

#### 2.2.12. Rheological Properties of Gels

The method of Kumar et al. [22] was modified. The measurements were carried out using a rheometer (HR20, Shanghai Sunny Hengping Scientific Instrument Co., Ltd., Shanghai, China). The temperature sweep used was as follows. First, 1.5 mL of gel was placed on the rheometer plate, and the rheological properties were analyzed using a plate–plate system (plate diameter 40 mm, gap 1000 μm). The temperature was then increased from 25 °C to 90 °C at a rate of 4 °C/min, held at 90 °C for 1 min for gelatinization, and the scanning frequency was 10 rad/s. The dynamic rheological properties included a frequency range of 0.1–100 Hz, and the test temperature was 25 °C. After determining the linear viscoelastic region (LVR) through strain scanning, it was found that 0.5% of the strain was completely within this linear region. The strain was set to 0.5%, and the storage modulus (G′), loss modulus (G″), and loss tangent (tanδ = G″/G′) were recorded as the angular frequency (ω) increased.

#### 2.2.13. Freeze–Thaw Stability of Gels

The method of Yuan et al. [23] was modified. The gels were frozen at −18 °C for 24 h, and then thawed at 25 °C for 6 h to complete one freeze–thaw cycle. This process was repeated for a total of 5 cycles. After each thawing cycle, the sample was centrifuged at 2000× *g* for 15 min (3H24R1, Changsha Xiangzhi Centrifuge Instrument Co., Ltd., Changsha, Hunan, China), and the separated water was collected and weighed. The syneresis rate of the gel was calculated according to the following formula:

(5) $$\begin{array}{c} syneresis\;rate\;\left(\%\right)\;=\;\frac{\left(m_{1}-m_{2}\right)}{m_{1}} \end{array}$$

where *m*_1_—the initial mass of the gel, g; and *m*_2_—the mass of the hydrogel after each freeze–thaw cycle, g.

#### 2.2.14. Color Difference Measurement of Breads

The L*, a*, and b* values of bread crust were measured using a color difference meter (DS-700D, Hangzhou Caipu Technology Co., Ltd., Hangzhou, Zhejiang, China). Before measurement, the instrument was calibrated with white cardboard. Different parts of each sample were measured three times, and the average values were calculated. The browning index (BI) for the crust was calculated as follows [24]:

(6) $$\begin{array}{c} BI\;=\;\frac{100\times (x-0.31)}{0.1712} \end{array}$$

(7) $$\begin{array}{c} x=\frac{a^{*}+1.75L^{*}}{5.645L^{*}+a^{*}-3.012b^{*}} \end{array}$$

#### 2.2.15. Texture Profile Analysis of Breads

The crusts and edges of the breads were removed, and the breads were cut into cubes of 3 cm × 3 cm × 2 cm. TPA was performed using a texture analyzer (TMS-pilot, Food Technology Corporation, Sterling, VA, USA) with a P/36 R probe (5 kg load cell) at a test speed of 1.0 mm/s, compression ratio of 50%, trigger force of 5 g, and distance of 40 mm. The hardness, springiness, and chewiness were recorded [25].

#### 2.2.16. Electronic Nose Analysis of Bread Aroma

The aroma characteristics of bread were determined using an electronic nose system (PEN3, Airsense Analytics GmbH, Schwerin, Germany). Briefly, 1 g bread sample was cut into pieces, placed in a headspace vial, and allowed to stand at room temperature for 30 min to enrich the headspace volatiles. The sensor cleaning time, analysis sampling time, zeroing time, sample preparation time, and injection flow rate were set at 120 s, 120 s, 5 s, 5 s, and 400 mL/min, respectively [26].

#### 2.2.17. Electronic Tongue Analysis of Breads

A total of 10 g of a sample was mixed with 50 mL of distilled water, homogenized using a blender, and then centrifuged at 4000× *g* for 10 min (3H24R1, Changsha Xiangzhi Centrifuge Instrument Co., Ltd., Changsha, Hunan, China). The supernatant was collected as the test liquid sample, and the taste profile of the bread was determined using an electronic tongue system (SA402B, Insent Inc., Atsugi, Japan). A nearly tasteless solution of potassium chloride (30 mM) and tartaric acid (0.3 mM) was used as the reference electrolyte solution. To ensure sensor stability, all sensors were soaked in the reference electrolyte solution for 24 h prior to the test. The acquired electrical signal data were processed to generate taste profiles corresponding to human taste perception [27].

#### 2.2.18. X-Ray Diffraction of Breads

The diffraction patterns of bread sample powders were measured using an X-ray diffractometer (Rigaku SmartLab SE, Rigaku Corporation, Tokyo, Japan) with Cu Kα radiation (λ = 1.54 Å). The voltage and current were set at 40 kV and 30 mA, respectively. The diffraction angle (2θ) was scanned from 5° to 35° at a scanning speed of 10°/min. GraphPad Prism 10 and Origin 2024 were used to plot the crystal type and crystallinity of the samples [22].

#### 2.2.19. Thermogravimetric Analysis (TGA) of Gels and Breads

Thermogravimetric analysis was performed using a thermogravimetric analyzer (TGA8000, PerkinElmer Management Co., Ltd., Shanghai, China). Briefly, 5 mg of bread samples were placed in a crucible, and the temperature was increased from 20 °C to 700 °C at a rate of 10 °C/min to evaluate the thermal stability of the samples [28]. The experiment was conducted under nitrogen (N_2_) at a flow rate of 20 mL/s. The mass loss of the sample as a function of temperature was recorded, and Origin 2024 was used for plotting.

#### 2.2.20. Determination of Water Loss Rate of Breads

After the bread cooled to room temperature, it was weighed, packaged in fresh-keeping bags, labeled, and stored at room temperature for 0, 1, 2, 3, 4, and 5 days. The weight was measured daily, and the water loss rate was calculated using the following formula:

(8) $$\begin{array}{c} water\;loss\;rate\;\left(\%\right)=\;\frac{\left(m_{1}-m_{2}\right)}{m_{1}}\times 100 \end{array}$$

where *m*_1_—the initial sample’s weight, g; and *m*_2_—the sample’s weight after storage, g.

#### 2.2.21. Metabolomics of Breads

The bread samples were subjected to simulated chewing treatment. Briefly, 5 g bread samples were vortex-mixed with 5 mL artificial saliva containing salivary amylase and incubated with shaking in a 37 °C air bath constant temperature oscillator (SHZ-82B, Changzhou Runhua Electrical Appliance Co., Ltd., Changzhou, Jiangsu, China) for 5 min [29]. Then, the digestive fluid was collected. After centrifugation (8000× *g*, 10 min), the supernatant was used for untargeted metabolomic analysis (Text S1). Data were analyzed by principal component analysis (PCA), differential metabolite analysis, sample correlation heatmap, and Venn diagram analysis.

### 2.3. Statistical Analysis

All experiments were performed at least three times, with data expressed as mean ± standard deviations, indicating error bars. Statistical significance was performed using SPSS 27 (Chicago, IL, USA) with Duncan’s multiple comparison test (*p* < 0.05), and graphs were plotted using Origin 2024 (Northampton, MA, USA) and GraphPad Prism 10 (La Jolla, CA, USA).

## 3. Results and Discussion

### 3.1. Basic Properties of Bamboo Shoot Superfine Powder and Structural Characteristics of the Bamboo Shoot Superfine Powder/κ-Carrageenan Composite Gel System

#### 3.1.1. Basic Properties of Bamboo Shoot Superfine Powder and Gel Properties

**Figure 1:**
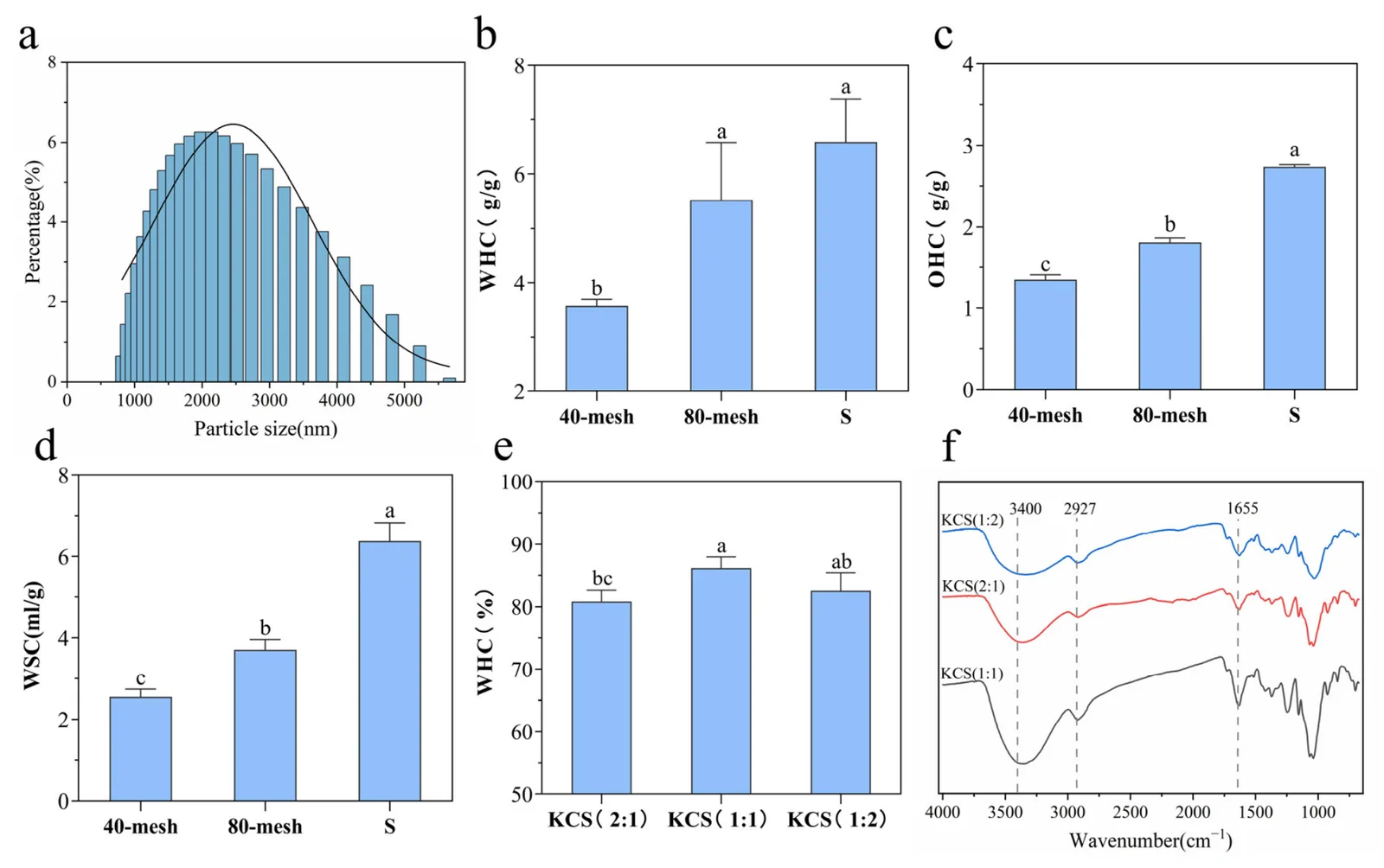
Basic properties of bamboo shoot superfine powder and gel properties. (**a**) Particle size distribution of bamboo shoot superfine powder; (**b**–**d**) WHCs, OHCs, and WSCs of bamboo shoot powders; (**e**) WHCs of gels with different blending ratios; (**f**) FTIR spectra of gels with different blending ratios. Different lowercase letters indicate significant differences between different data points, *p* < 0.05.

The basic composition of bamboo shoot shells is shown in Table S1. The total dietary fiber content of bamboo shoot shells was 49.89%, with 24.72% cellulose, 14.92% hemicellulose, and 23.03% lignin. In addition, the bamboo shoot shell has the characteristics of being high in protein (15.7%) and low in fat (0.8%). Furthermore, a superfine powder generally refers to a material with particle sizes below 1–10 μm. The bamboo shoot superfine powder exhibited a D50 of 1578.74 nm and a D90 of 2105.28 nm (Figure 1a). The WHCs, OHCs, and WSCs of 40-mesh bamboo shoot powder, 80-mesh bamboo shoot powder, and bamboo shoot superfine powder were measured separately (Figure 1b–d). All three parameters increased with decreasing particle size, suggesting that superfine grinding improved the hydration and oil-binding properties of bamboo shoot powder. This enhancement was primarily attributed to the increased specific surface area, which facilitates the interaction of water and oil molecules with the particles, as well as the disruption of the compact dietary fiber matrix, leading to a more porous structure and greater exposure of hydrophilic and lipophilic functional groups that promote water and oil adsorption. Zhang et al. [30] modified the insoluble dietary fiber from soybean skins with penicillium cellulase. After modification, the specific surface area significantly increased, and the water-retention capacity and oil-retention capacity both improved significantly [30]. The water-holding capacity of the composite gels varied with the mixing ratio (Figure 1e), with the KCS (1:1) group showing the highest value. This suggests that, at this ratio, the bamboo shoot superfine powder was more uniformly dispersed within the κ-carrageenan network, facilitating the formation of a denser gel matrix that effectively restricted water mobility and reduced water loss. The FTIR spectra of all samples exhibited a broad absorption peak near 3400 cm^−1^, mainly due to O–H stretching vibrations. Compared with KCS (1:1), both KCS (2:1) and KCS (1:2) showed lower peak intensities and peak shifts, while the absorption peak of KCS (1:1) became broader, which may be due to enhanced hydrogen bond formation. In addition, weak absorption peaks near 2927 cm^−1^ and 1655 cm^−1^ were observed in all samples, related to C–H and –COOH stretching vibrations, suggesting that, after compounding bamboo shoot powders with κ-carrageenan, hydrophobic interactions or molecular chain rearrangement might exist. In summary, KCS (1:1) was the optimal group for forming a new composite gel network.

#### 3.1.2. Structural Characteristics of the Bamboo Shoot Superfine Powder/κ-Carrageenan Composite Gel System

According to Section 3.1.1, KCS (1:1) was the optimal formulation for forming the novel composite gel network. Hereafter, KCS denotes the composite gel of bamboo shoot superfine powder and κ-carrageenan at a 1:1 ratio, and the properties of κ-carrageenan gel (KC), bamboo shoot superfine powder (S), and κ-carrageenan and bamboo shoot superfine powder composite gel (KCS) were compared and analyzed.

**Figure 2:**
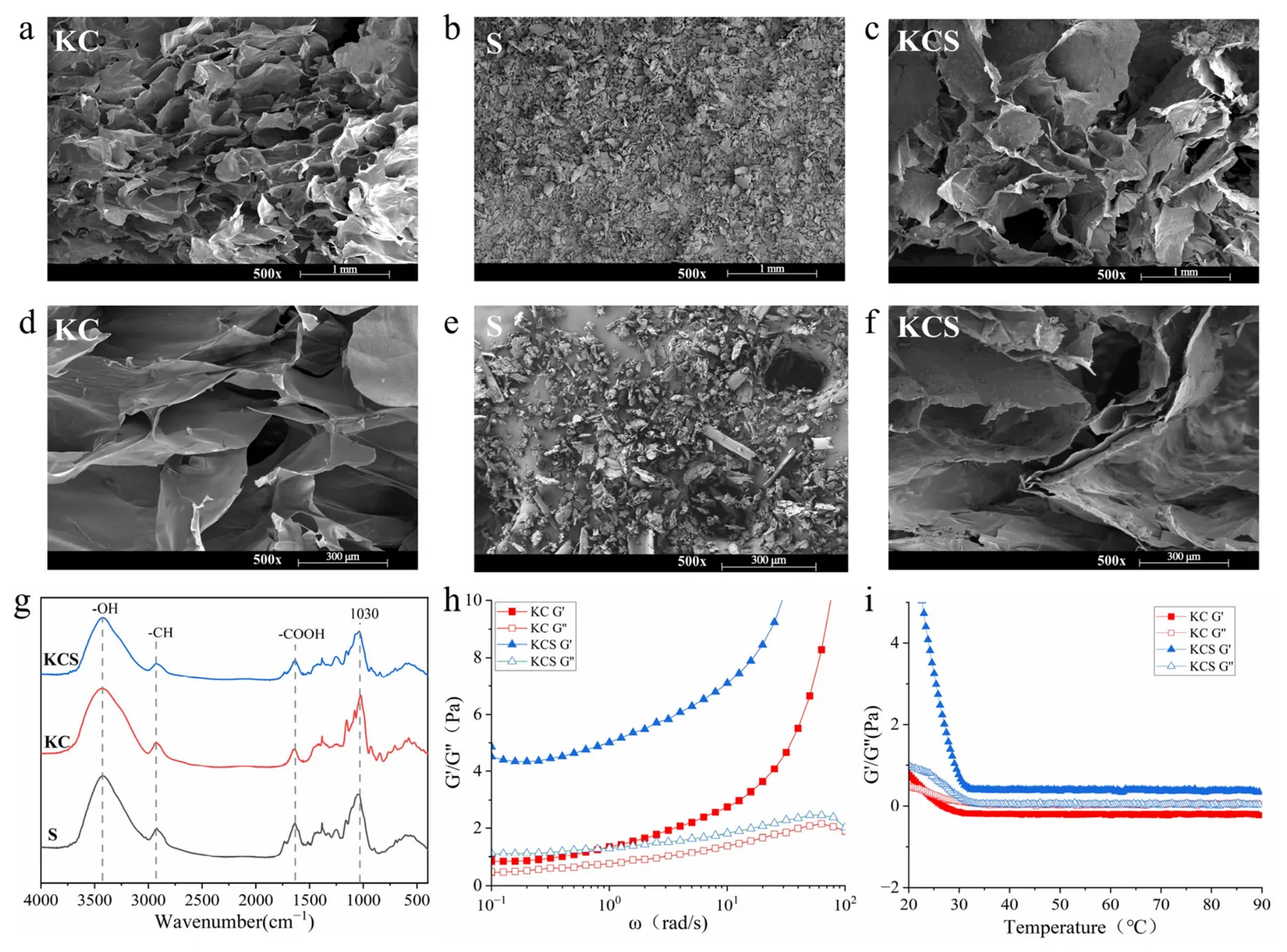
Structural characteristics of different composite gels. (**a**–**f**) Scanning electron microscope images of different composite gels; (**g**) FTIR spectra of different composite gels; (**h**,**i**) rheological properties of different composite gels. κ-carrageenan gel (KC), bamboo shoot superfine powder (S), and κ-carrageenan and bamboo shoot superfine powder composite gel (KCS).

Scanning electron microscopy was used to observe the microstructures of different materials. The KC group exhibited a smooth, continuous flaky structure with a few unevenly distributed pores (Figure 2a,d), giving the gel a certain rigidity and brittleness while limiting its textural properties [31]. The S group exhibited a rough surface morphology. Following superfine grinding, the particle size was markedly reduced, resulting in irregularly shaped particles that were loosely packed with numerous voids (Figure 2b,e). Compared with KC and S, the KCS group showed a denser, more continuous composite network structure (Figure 2c,f). The continuous network of KC provided the overall framework of the gel, and the embedding of bamboo shoot powder particles effectively filled the network pores, making the entire structure appear denser. This result is similar to the findings of Wu et al. [9]. They proposed that insoluble citrus fiber (ICF) acts as a filler embedded in the κ-carrageenan (KC) gel matrix, and hydrogen-bonding interactions between ICF and KC lead to the formation of a denser network structure.

FTIR spectroscopy is an effective means of studying intermolecular interactions and functional group changes. Absorption peaks at 3400 cm^−1^, 2920 cm^−1^, and 1625 cm^−1^ represent O–H stretching vibrations, C–H stretching vibrations, and C=O bending vibrations, respectively [32]. All spectra showed a strong, sharp absorption peak near 3400 cm^−1^, mainly due to O–H stretching (Figure 2d). The KCS group exhibited a broadened peak and slight wavenumber shift, likely due to the formation of intermolecular hydrogen bonds between κ-carrageenan and cellulose, hemicellulose, and other substances in the bamboo shoot superfine powder [33]. Additionally, a weak absorption peak at 1625 cm^−1^ (C=O bending) was observed, which broadened in the KCS group, suggesting that carboxyl groups in bamboo shoot superfine powder might undergo ionic interactions with the sulfate or hydroxyl groups of κ-carrageenan. A weak band near 2920 cm^−1^, assigned to polysaccharide C–H stretching vibrations, was observed in all samples. The broadening of the absorption peak of KCS at 1030 cm^−1^ indicated that the introduction of the bamboo shoot superfine powder disrupts the original ordered structure of κ-carrageenan, while the dietary fibers form new hydrogen bonds with the κ-carrageenan molecular chains. This synergistic effect of physical crosslinking and hydrogen bonding facilitates the reconstruction of the composite gel network and promotes its densification [34]. The S and KCS groups had similar absorption peaks without new peaks, indicating that no new functional groups were generated during gel formation. These results indicated that S and KC formed a composite gel network through non-covalent interactions, primarily hydrogen bonding, with electrostatic interactions providing additional stabilization [35].

Figure 2h showed the frequency sweep of the two gels. For both gels, G′ remained higher than G″ across the tested frequency range, with no crossover between the two curves, confirming the formation of stable elastic gel networks. Notably, the KCS gel exhibited a higher G′ value than KC, indicating enhanced elasticity. This enhancement is likely attributable to the incorporation of dietary fiber-rich bamboo shoot superfine powder as a rigid filler within the κ-carrageenan network, thereby increasing network density and mechanical strength [9]. The temperature dependence of G′ and G″ for the two gels is shown in Figure 2i. With increasing temperature, both KC and KCS exhibited a decrease in G′ and G″, followed by relatively stable values at higher temperatures. KCS showed a more pronounced decrease in G′ at lower temperatures but maintained G′ > G″ throughout the tested range (20–90 °C), indicating the retention of a predominantly elastic, gel-like rheological response without a detectable gel–sol transition. In contrast, the G′ of KC remained much lower and approached the instrumental detection limit at elevated temperatures, reflecting its weaker elastic network. Overall, KCS exhibited greater resistance to thermal weakening than KC under the tested conditions, possibly due to additional intermolecular interactions that help maintain the gel network at elevated temperatures. In summary, dietary fibers in bamboo shoot superfine powders can form a new composite gel network structure through hydrogen-bonding and non-covalent interactions, synergistically enhancing the mechanical properties and thermal stability of κ-carrageenan gel and showing good potential for application in thermally processed foods.

#### 3.1.3. Stability Analysis of the Bamboo Shoot Superfine Powder/κ-Carrageenan Composite Gel System

**Figure 3:**
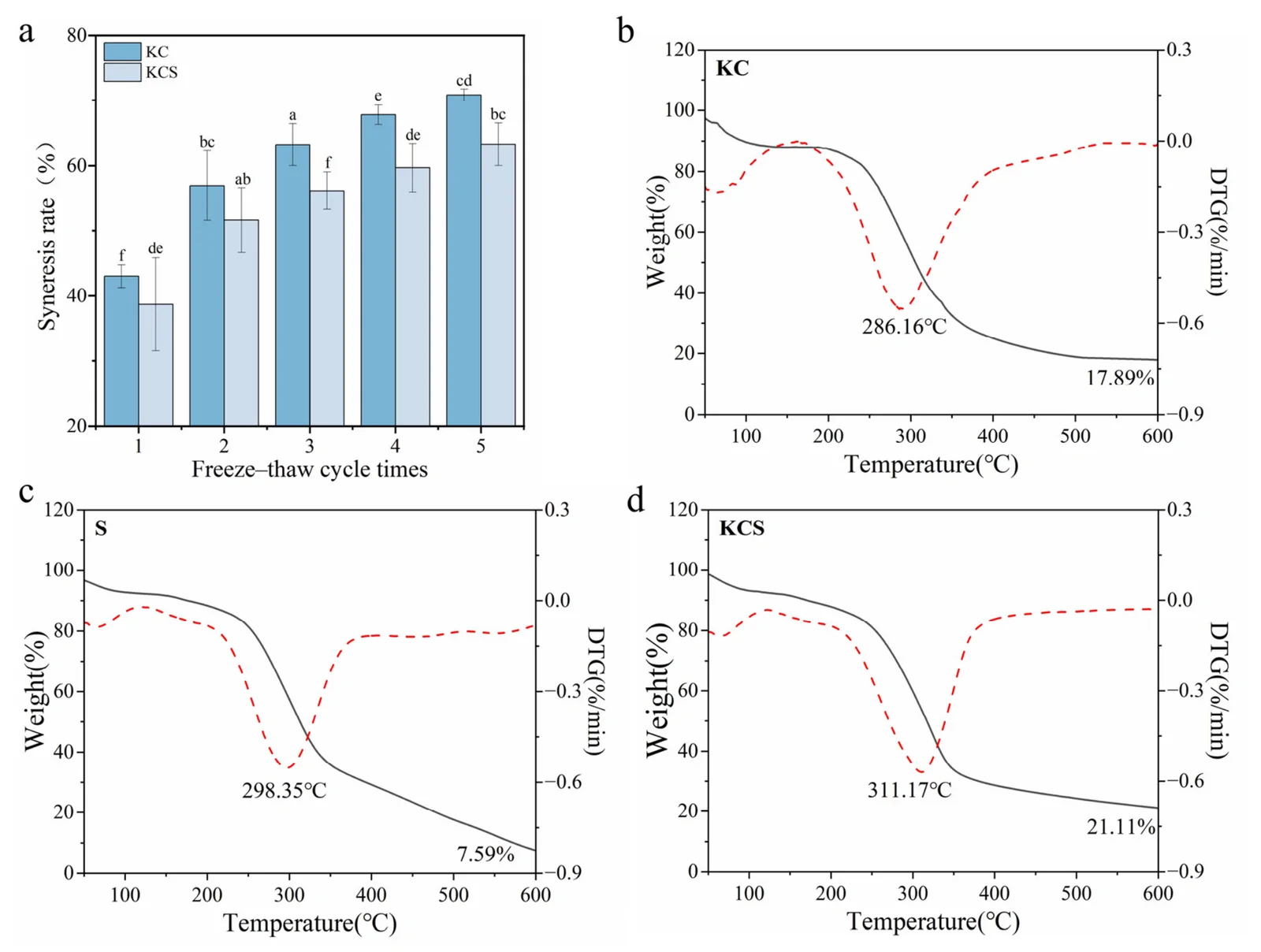
Stability analysis of different composite gels. (**a**) Syneresis rate during freeze–thaw cycles; (**b**–**d**) thermogravimetric analysis curves. κ-carrageenan gel (KC), bamboo shoot superfine powder (S), and κ-carrageenan and bamboo shoot superfine powder composite gel (KCS). The black curve is the TG curve, and the red curve is the DTG curve. Different lowercase letters indicate significant differences between different data points, *p* < 0.05.

Freeze–thaw stability can be employed to characterize the stability of gel systems. During the freezing process, the phase transition of freezable water (including free water and freezable bound water) induces potential disruption or damage to the polymer network through the uncontrolled growth of ice crystals [36]. Upon thawing, water separates from the gel, leading to a marked deterioration in product quality. As shown in Figure 3, the syneresis rate of all hydrogels increased progressively with the number of freeze–thaw cycles. Notably, KC consistently exhibited a higher syneresis rate than KCS, indicating that the pure κ-carrageenan network was more susceptible to ice-crystal-induced structural damage, resulting in greater water loss during thawing. The incorporation of bamboo shoot superfine powder significantly enhanced the freeze–thaw stability of the hydrogels. The powder was uniformly dispersed within the κ-carrageenan matrix and embedded into the gel network, forming a composite gel with higher crosslinking density, thereby inhibiting water exudation.

To further investigate the effects of bamboo shoot superfine powders on the thermal stability of gels, thermogravimetric analysis (TGA) was performed (Figure 3b–d). The TGA curves indicated three-stage thermal degradation for all samples. The first stage (30–260 °C) was associated with the evaporation of free and bound water, whereas the major degradation occurred at 260–320 °C. Notably, the peak degradation temperature of KCS increased by 25.01 °C compared with KC. This improvement was attributed to the enhanced hydrogen bonding induced by bamboo shoot superfine powders, which promoted a more compact gel network and improved thermal stability. The third stage (320–600 °C) corresponded to carbonization, involving polysaccharide backbone scission and the decomposition of refractory components, such as lignin. At the end of the heating process, the residual mass of KCS at 600 °C was 3.22% higher than that of KC, further confirming that the bamboo shoot superfine powder effectively enhances the thermal stability of the composite gel.

### 3.2. Basic Performance Analysis of Bread with Bamboo Shoot Superfine Powder/κ-Carrageenan Composite Gel

#### 3.2.1. Color Parameters and Texture Properties

**Figure 4:**
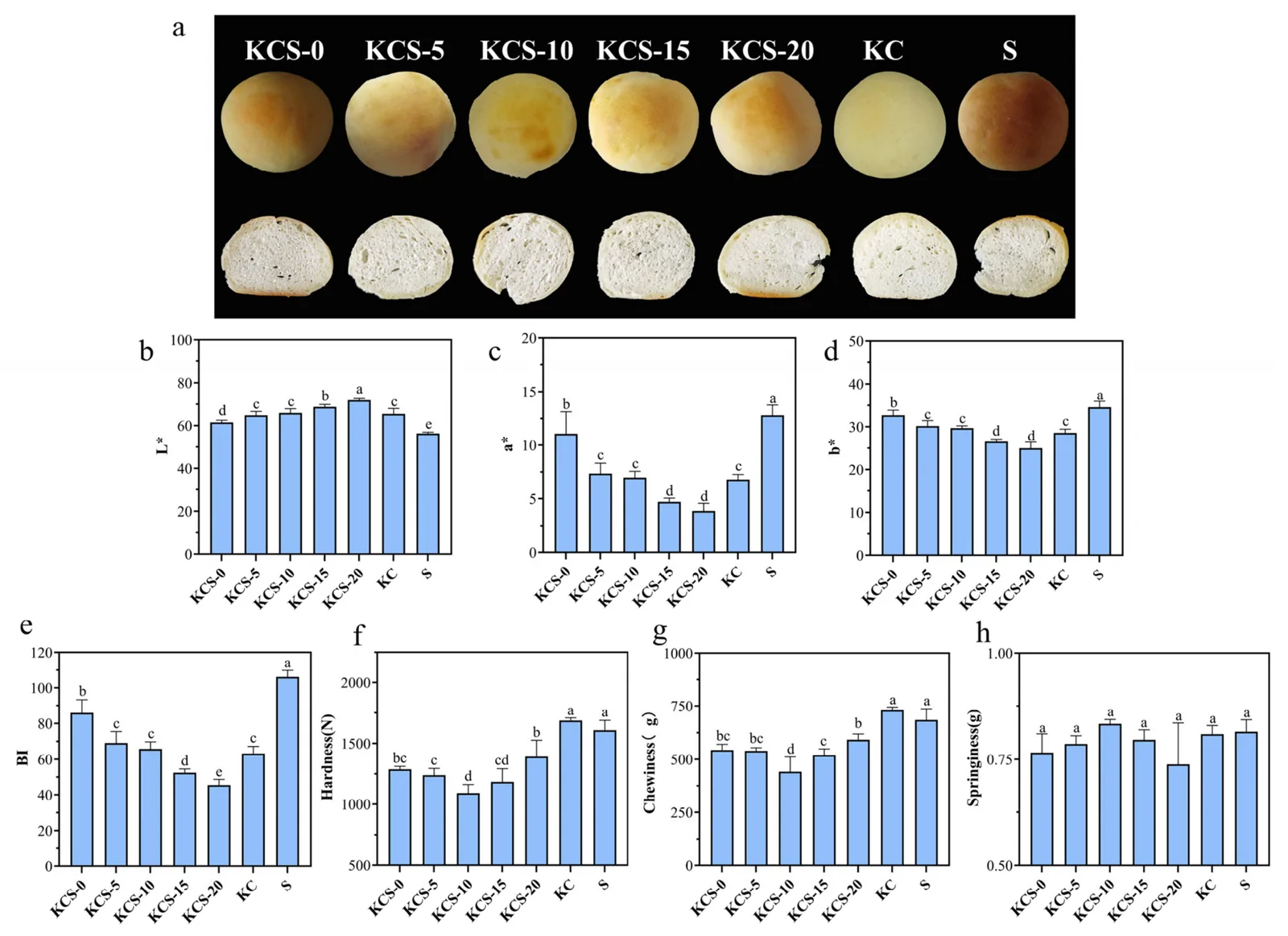
Color parameters and textural properties of different breads. (**a**) Photographs of the surface appearance and internal cross-sectional structure of breads; (**b**) L*; (**c**) a*; (**d**) b*; (**e**) BI; (**f**) hardness; (**g**) chewiness; (**h**) springiness. Bread with pure κ-carrageenan gel (KC); bread with bamboo shoot superfine powder (S); bread without any addition (KCS-0); breads with the additions of 5%, 10%, 15%, and 20% of the bamboo shoot superfine powder composite gel (KCS-5, KCS-10, KCS-15, and KCS-20). Different lowercase letters indicate significant differences between different data points, *p* < 0.05.

A color difference meter was used to quantitatively analyze the color of bread with different additions. Figure 4a shows the appearance and cross-sectional photographs of different breads. The L* value represents brightness, the a* value represents redness-greenness, and the b* value represents yellowness–blueness (Figure 4b–d). Compared with the control, the KC group exhibited a higher L* value and lower a* and b* values, indicating a brighter appearance. In contrast, the S group showed a lower L* value and higher a* and b* values, resulting in a darker, more yellowish color. This result may be attributed to the significant increase in the contents of polysaccharides and soluble dietary fibers in bamboo shoot powders caused by the superfine grinding treatment (Table S1). Under the high temperatures of baking, these components can be partially hydrolyzed to generate low-molecular-weight reducing sugars, such as glucose and fructose, which directly provide more abundant carbonyl donors for the Maillard reaction. In addition, the enrichment of dietary fibers alters the water status and its migration behavior in the bread system, and the consequent reduction in available water enhances the formation of later-stage Maillard reaction products. Together, these factors promote the Maillard reaction during baking [37,38]. Fatima et al. [39] found that okra polysaccharides affect the color of wheat bread dough. For bread with the composite gel (KCS), as the addition level increased, L* gradually increased while a* and b* gradually decreased. The BI value is an objective indicator that quantifies the browning degree of bread crusts. Its core role is to reflect the extents of the Maillard reaction and caramelization during the baking process. As the added amount of composite gel increases, the BI value decreases, resulting in a lighter bread color, among which the bread in the S group showed the deepest color (Figure 4e). The bread became brighter, and its red and yellow values were decreased, indicating that the composite gel increased brightness via κ-carrageenan and utilized the characteristic color of bamboo shoot powders, avoiding the monotonous color of κ-carrageenan alone and the overly dark color of bamboo shoot powder alone.

Texture properties are important indicators for evaluating bread quality. Wang et al. [40] found that insoluble dietary fibers can hinder the formation of the gluten network through steric hindrance, ultimately leading to the deterioration of steamed bread quality. The hardnesses of the KC and S groups were significantly higher than that of the blank group (Figure 4f). As a hydrophilic colloid, κ-carrageenan competes with starch and gluten proteins for available water during dough formation. Upon cooling, it undergoes coil-to-helix transition and subsequently forms a continuous and rigid gel network. In bread with S treatment, the increased dietary fibers lack the extensibility and viscoelasticity of gluten proteins, and the rigid fiber structure disrupts the gluten network, thereby increasing hardness and chewiness. For the KCS group, hardness and chewiness first decreased and then increased, showing no significant difference from the blank group and being overall lower than the KC and S groups (Figure 4f,g). This likely occurs as the fibers in the bamboo shoot superfine powder integrate into the κ-carrageenan gel network, creating a composite gel matrix that retains water and maintains structural stability. This trend was consistent with previous studies on dietary fiber-fortified bread systems. The addition of insoluble dietary fiber has been reported to significantly increase bread hardness and chewiness [41]. Springiness is the ability of a sample to recover its original shape after the first compression. As shown in Figure 4h, no significant differences in springiness were observed among the bread groups. In summary, an appropriate amount of bamboo shoot superfine powder/κ-carrageenan composite gel helps improve the color and texture of bread, optimizing its palatability and potentially increasing consumer sensory acceptance.

#### 3.2.2. Electronic Nose and Electronic Tongue Analyses

**Figure 5:**
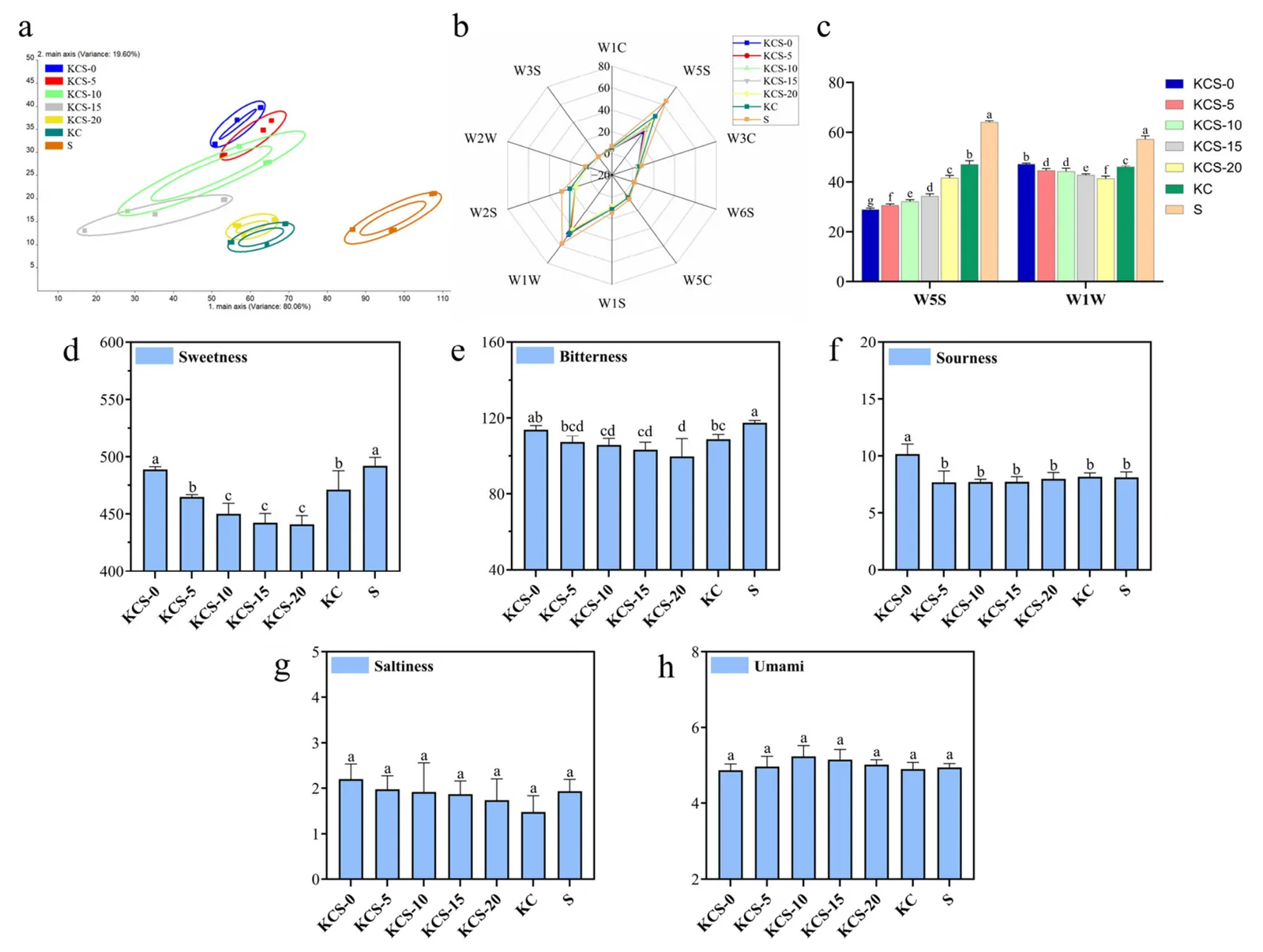
Flavor analysis of different breads. (**a**) PCA diagram; (**b**) electronic nose radar diagram; (**c**) characteristic flavor comparison; (**d**–**h**) electronic tongue flavor comparison. Bread with pure κ-carrageenan gel (KC); bread with bamboo shoot superfine powder (S); bread without any addition (KCS-0); breads with the additions of 5%, 10%, 15%, and 20% of the bamboo shoot superfine powder composite gel (KCS-5, KCS-10, KCS-15, and KCS-20). Different lowercase letters indicate significant differences between different data points, *p* < 0.05.

Electronic nose analysis combined with principal component analysis (PCA) was used to evaluate differences in bread aroma profiles. As shown in Figure 5a, PC1 and PC2 accounted for 80.06% and 19.60% of the total variance, respectively, explaining 99.66% of the overall variation. These results indicated that the PCA model effectively captured the aroma characteristics of the bread samples. The flavor profiles of the KC and S groups differed greatly from the blank group, indicating they had different volatile gas compositions. The KCS group had volatile gas compositions closer to the blank group. Based on the electronic nose sensor response values, an odor radar map was constructed (Figure 5b). The ten sensors represent aromatic components (W1C); nitrogen oxides (W5S); ammonia and aromatic compounds (W3C); hydrogen (W6S); alkanes and weakly polar compounds (W5C); methane and short-chain alkanes (W1S); inorganic sulfides (W1W); alcohols, aldehydes, ethers, and similar substances (W2S); aromatic compounds and organic sulfides (W2W); and alkanes (W3S). The odor profiles of the seven samples were similar, with little change in the response values of sensors W1C, W3C, W6S, W5C, W1S, W2W, and W3S. As shown in Figure 5c, the responses of two sensors with significant changes, W5S and W1W, were further analyzed. W5S detects nitrogen-oxide-like volatile components in bread, primarily contributing baked, nutty, and cereal-toasted notes. Too low a content results in a bland flavor lacking a baked note, while too high a content quickly turns the baked aroma into a burnt, bitter taste [42,43]. After adding the composite gel, W5S significantly increased, and with increasing additions of gel, W5S tended to rise. This may be due to the amino acids and reducing sugars in bamboo shoot superfine powders enhancing the Maillard reaction under high-temperature baking, generating more flavor compounds while possibly producing by-product nitrogen oxides. The KC and S groups showed a substantial increase in W5S, negatively affecting the bread aroma by turning the baked note into a burnt, off-flavor [43]. W1W detects inorganic sulfides, which impart sulfurous, roasted, and slightly pungent spicy notes [43]. The significantly lower W1W response in the KCS group suggests that the three-dimensional network formed by the bamboo shoot superfine powder and κ-carrageenan effectively retained volatile sulfur compounds, limiting their migration into the gas phase and thereby reducing the release of undesirable sulfur-related odors.

Taste is an important component of food flavor, directly reflecting the taste and flavor of the product. An electronic tongue uses electronic sensors to analyze, identify, and judge the overall taste of samples, mimicking the human tongue [44]. The results showed that the addition of KCS composite gel significantly affected the sweetness, sourness, and bitterness of bread by reducing its sourness, sweetness, and bitterness (Figure 5d–h). Notably, sweetness decreased progressively with increasing composite gel addition, suggesting that the bamboo shoot superfine powder/κ-carrageenan network restricted the diffusion and release of low-molecular-weight flavor compounds. Overall, the composite gel effectively modulated the flavor release, suppressing undesirable volatile compounds while enhancing aroma complexity and improving the sensory quality of bread.

### 3.3. Structural Characteristics of Bread with Bamboo Shoot Superfine Powder/κ-Carrageenan Composite Gel

#### 3.3.1. Microstructure and Molecular Structure

**Figure 6:**
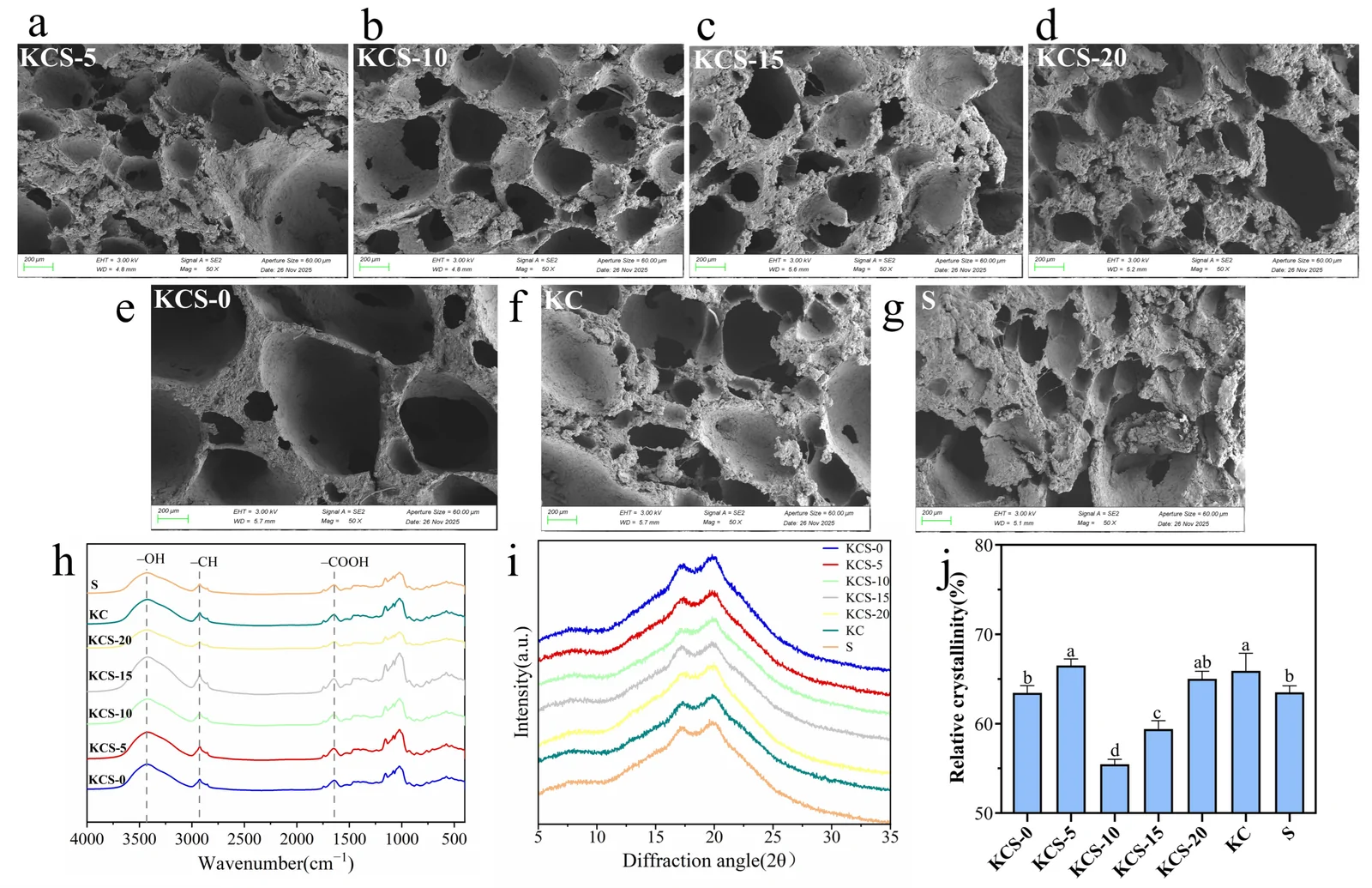
Microstructures and molecular structures of different breads. (**a**–**g**) Scanning electron microscope images of different breads; (**h**) FTIR spectra of different breads; (**i**) X-ray diffraction patterns of different breads; (**j**) relative crystallinity of different breads. Bread with pure κ-carrageenan gel (KC); bread with bamboo shoot superfine powder (S); bread without any addition (KCS-0); breads with additions of 5%, 10%, 15%, and 20% of the bamboo shoot superfine powder composite gel (KCS-5, KCS-10, KCS-15, and KCS-20). Different lowercase letters indicate significant differences between different data points, *p* < 0.05.

Scanning electron microscopy can reveal the microstructures of samples and help investigate the interaction mechanisms between bamboo shoot superfine powder, starch, and gluten proteins, which are key internal factors determining bread quality [45]. The gluten network structure is closely related to bread quality. The KCS-0 group showed a smooth, flat surface with large, evenly distributed pores and a continuous and uniform albeit relatively loose gluten network (Figure 6e). After baking, the bread was soft but had poor chewiness. In the KC and S groups, the gluten network structure was disrupted, with broken network segments and a rough surface (Figure 6f,g). This phenomenon is closely related to κ-carrageenan and insoluble dietary fibers competing with gluten proteins for the available water, which prevents the formation of a continuous film-like gluten structure. Moreover, the hard bamboo shoot powder particles disrupted the gluten network during fermentation, which was consistent with the findings of Liu et al. [46]. The KCS group exhibited a denser porous structure (Figure 6a–d). At 10% composite gel addition, a uniform, fine honeycomb network structure appeared, resulting in bread with good chewiness and springiness after baking. κ-carrageenan encapsulates the bamboo shoot superfine powder to form a composite gel network, reducing the damage of high-fiber raw materials to the gluten network. As the addition level further increases, pore wall rupture and structural collapse with irregular pores occur, possibly due to the excessive composite gel network squeezing and disrupting the continuity of the gluten protein structure.

As shown in Figure 6h, all FTIR spectra showed a strong absorption peak near 3400 cm^−1^, mainly due to O–H and N–H stretching vibrations. The S group exhibited a broadened absorption peak, likely due to the abundant hydroxyl groups in bamboo shoot superfine powders that interact with water through hydrogen bonding. The KC group showed enhanced peak intensity, reflecting the strong water-binding capacity of κ-carrageenan via its hydroxyl and sulfate groups. In the KCS group, the absorption peak intensity increased initially and then decreased with increasing composite gel addition. KCS-10 had a moderate absorption peak intensity near 3400 cm^−1^, indicating that κ-carrageenan and dietary fibers in the bamboo shoot superfine powder crosslinked through hydrogen bonds to form a composite gel network that was more ordered and stable. The amide I band (1600–1700 cm^−1^) is commonly used to study the secondary structures of proteins, mainly reflecting the C=O stretching vibrations of peptide bonds [47]. The KC group had a similar peak shape to KCS-0, indicating that κ-carrageenan had little effect on the protein secondary structure. The S group showed reduced absorption peak intensity in this region, possibly because dietary fibers compete with gluten proteins for water, causing protein aggregation and conformational changes, which leads to decreased gluten network extensibility [48]. With increasing composite gel additions, the absorption peak intensity first increased and then decreased in 1600–1700 cm^−1^. This result suggests that an appropriate amount of the S and KC composite gel helps stabilize the conformation of gluten proteins and promotes the formation of ordered secondary structures in breads.

X-ray diffraction is an effective means of studying the crystalline structure of starch, reflecting crystal transformation and crystallinity changes during processing. Starch retrogradation is essentially the transition of gelatinized starch molecules from an amorphous state to an ordered crystalline state, which also affects bread texture [49]. As shown in Figure 6i, all bread samples exhibited similar XRD patterns, with characteristic starch crystallinity peaks at 17°and 20°. The V-type crystalline peak at 2θ = 20° represents the amylose–lipid complex [50], which was present in all samples, indicating that the composite gel did not alter the starch crystal structure. In addition, the KC and S groups showed increased relative crystallinity, indicating that κ-carrageenan and superfine bamboo shoot powders promoted the ordered arrangement of amylose. Compared with the blank group, the relative crystallinity of the KCS group first decreased and then increased, reaching the lowest value at 10% composite gel addition, which was 7.99% lower than that of the blank group. This indicates that an appropriate amount of composite gel inhibits starch retrogradation and reduces crystallinity, possibly because the composite gel network hinders the diffusion and ordered arrangement of starch molecular chains. At higher addition levels, crystallinity increased, likely because the excessive composite gel disrupted the gluten network and promoted starch retrogradation, consistent with the SEM observations. As a major contributor to bread staling, starch retrogradation is associated with increased hardness and reduced springiness, in agreement with the texture analysis. Wang et al. [51] found that buckwheat hull at the cell scale had a stronger water-binding ability than tissue-scale buckwheat hull, which restricted the loss of water, inhibiting bread staling. In summary, an appropriate amount of KCS in bread processing helps form a stable, ordered gluten protein network structure, inhibit starch retrogradation, and improve bread quality.

#### 3.3.2. Thermal Property Analysis

**Figure 7:**
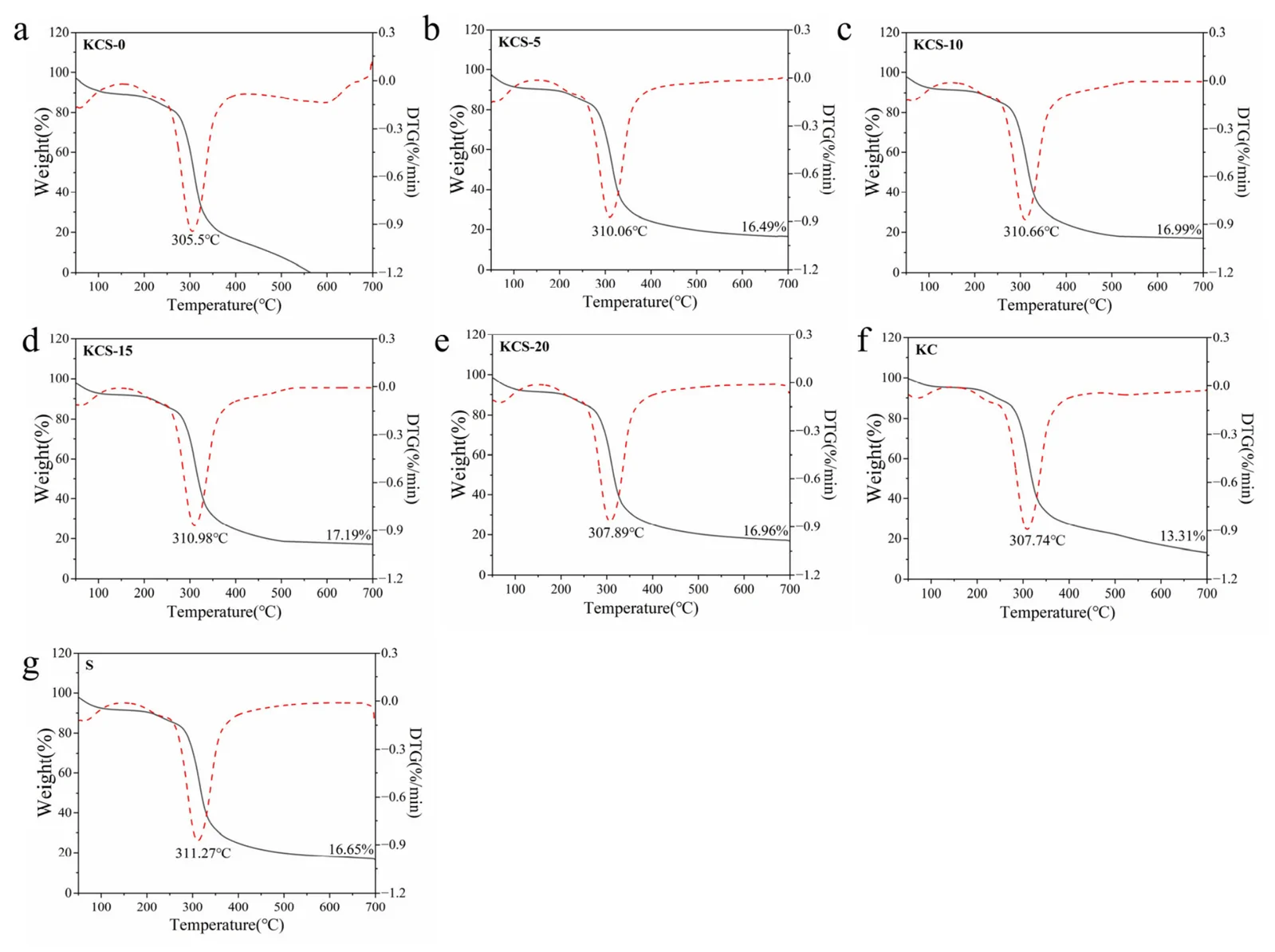
Thermogravimetric analysis curves of different breads. (**a**) KCS-0; (**b**) KCS-5; (**c**) KCS-10; (**d**) KCS-15; (**e**) KCS-20; (**f**) KC; (**g**) S. Bread with pure κ-carrageenan gel (KC); bread with bamboo shoot superfine powder (S); bread without any addition (KCS-0); breads with additions of 5%, 10%, 15%, and 20% of the bamboo shoot superfine powder composite gel (KCS-5, KCS-10, KCS-15, and KCS-20). The black curve is the TG curve, and the red curve is the DTG curve.

As shown in Figure 7, the TG and DTG curves of all bread samples were studied by TGA. All samples exhibited multi-step thermal degradation, which could be divided into three stages. The first stage (50–270 °C) mainly involved the evaporation of free water. The second stage (270–380 °C) showed the largest weight loss, associated with starch degradation and the degradation of polysaccharides, cellulose, and hemicellulose in the composite gel [52]. The third stage (380–700 °C) was mainly associated with the carbonization of residual components. Compared with the blank group, all supplemented bread samples exhibited a higher residual mass at 700 °C, with the KCS groups showing greater residues than the KC and S groups. Among them, KCS-20 had the highest residues. With increasing KCS addition, the residues first increased and then decreased. The peak temperature of the DTG curve reflects the thermal stability of the starch sample, also known as the decomposition temperature [53]. As shown in Figure 7, S exhibits a higher peak temperature, likely due to the dietary fiber content in bamboo shoot superfine powders, which enhances the thermal stability of the sample. The peak temperature of bread with composite gel increased; specifically, the peak temperature of KCS-15 was 5.48 °C higher than that of the blank group KCS-0. These results suggest that the bamboo shoot superfine powder/κ-carrageenan composite gel enhances the thermal stability of the bread matrix. This effect is likely attributable to the formation of a denser network structure with starch, which restricts molecular mobility and increases the energy required for thermal decomposition. Consequently, higher decomposition temperatures and residual masses were observed. Overall, an appropriate level of composite gel improved the thermal stability of bread.

### 3.4. Storage Stability of Bread with Bamboo Shoot Superfine Powder/κ-Carrageenan Composite Gel

**Figure 8:**
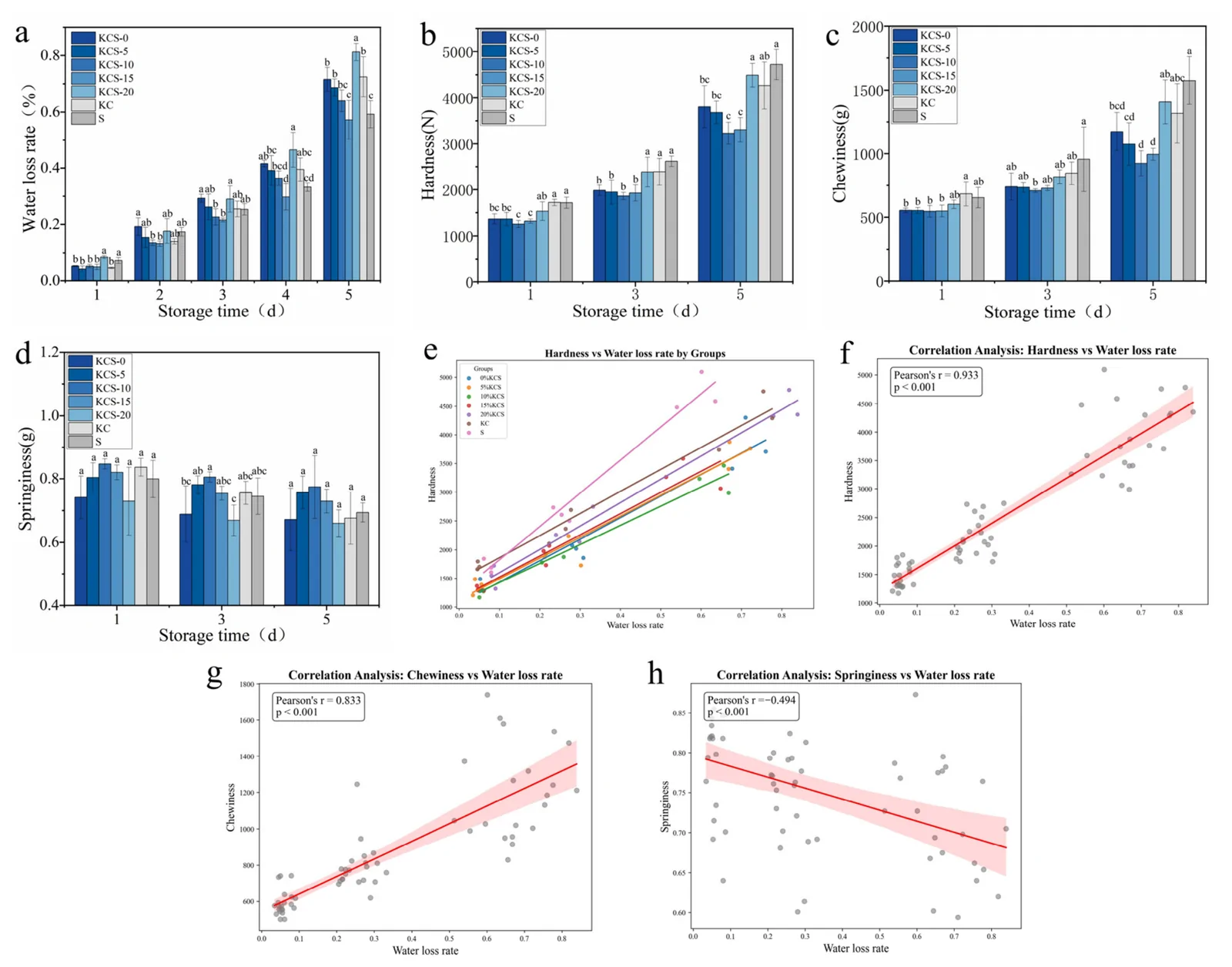
Storage properties of different breads. (**a**) Water loss rate; (**b**–**d**) textural property changes; (**e**–**h**) Pearson correlation analysis. Bread with pure κ-carrageenan gel (KC); bread with bamboo shoot superfine powder (S); bread without any addition (KCS-0); breads with additions of 5%, 10%, 15%, and 20% of the bamboo shoot superfine powder composite gel (KCS-5, KCS-10, KCS-15, and KCS-20). Different lowercase letters indicate significant differences between different data points, *p* < 0.05.

Moisture content is a key factor affecting bread quality and storage characteristics. During storage, the water loss rate of all samples increased (Figure 8a). Compared with the blank group, the water loss rate of the S group gradually decreased, as dietary fibers in bamboo shoot superfine powder can bind water molecules via hydrogen bonds, hindering water migration [54]. The KC group showed a higher water loss rate than the S group in the later storage period, indicating that the water-holding capacity of κ-carrageenan in bread was weaker than that of bamboo shoot superfine powder. For the KCS group, the water loss rate first decreased and then increased with its increasing addition; KCS-10 and KCS-15 had the lowest water loss rates and the best water-holding capacities during storage. As shown in Figure 8a, the water loss rate of KCS-20 was higher than that of KCS-15. Gluten protein is the key to forming a porous structure and retaining moisture in bread. When the gluten network is disrupted, it cannot effectively bind water, leading to a decline in the water-holding capacity during storage [55]. Excessive addition of KCS competes with gluten for the limited water in the system, hindering the full hydration of gluten and the formation of a strong and resilient network. This results in a reduced water-holding capacity, a loose structure, and uneven porosity, which are consistent with the texture analysis results. In the late-storage period, the water loss rate accelerated, which is closely related to starch retrogradation. During storage, moisture migrates from the crumb to the crust and is progressively lost, while amylose recrystallization further reduces the amount of free water [56,57].

Bread staling is the main quality deterioration during storage. Textural properties are the most direct indicators for evaluating the degree of staling. Changes in hardness, chewiness, and springiness of bread after storage for 1, 3, and 5 d were shown in Figure 8b–d. As shown in Figure 8b,c, hardness and chewiness increased with the storage time. The KC and S groups consistently had higher hardness and chewiness. The rigid gel network formed by κ-carrageenan further contracted and hardened with extended storage, while the addition of superfine bamboo shoot powders hindered the formation of the gluten network, reducing dough extensibility. These structural defects accelerated staling in the later storage period, with rapid increases in hardness and chewiness. For the KCS group, hardness and chewiness first decreased and then increased with its increasing addition. During storage, the KCS group showed a slower increase in hardness and chewiness, effectively inhibiting bread staling, with 10% addition being the most effective. Furthermore, as shown in Figure 8d, the springiness of all samples slightly decreased with storage time, and KCS-10 exhibited the best springiness, indicating that the composite gel more effectively slowed down bread staling and improved storage stability.

Pearson correlation analysis (Figure 8e–h) showed that during 5 days of storage, the water loss rate of bread was extremely significantly positively correlated with hardness (r = 0.933, *p* < 0.001), significantly positively correlated with chewiness (r = 0.833, *p* < 0.001), and negatively correlated with springiness (r = −0.494, *p* < 0.001). These results were consistent with the findings of Gao et al. on the changes in the water loss rate and texture of steamed bread during frozen storage, who reported that the water loss rate increased along with significant increases in hardness and chewiness and a decrease in springiness [58]. According to Figure 8e and Table S2, the rate at which hardness increased with the water loss rate varied significantly between breads with different additions. The slope was highest for group S (5777.1230) and lowest for group KCS-10 (3322.0901), indicating that KCS-10 had the best anti-staling ability. The bamboo shoot superfine powders possesses an excellent water-holding capacity. Zhang et al. [54] showed that the water-holding capacity of bamboo shoot dietary fibers was significantly better than that of rice and soybean dietary fibers, and could significantly improve the viscoelasticity and extensibility of frozen dough, as well as enhance the water retention of the dough system by altering the water distribution. In addition, κ-carrageenan and polysaccharide molecules can significantly improve gel strength and water-holding capacities by enhancing hydrogen bonding and forming three-dimensional network structures [59]. This study demonstrated that KCS effectively restricted water migration and delayed bread staling and texture deterioration during storage, with KCS-10 exhibiting the most pronounced anti-staling effect.

### 3.5. Metabolomics of Bread with Bamboo Shoot Superfine Powder/κ-Carrageenan Composite Gel

**Figure 9:**
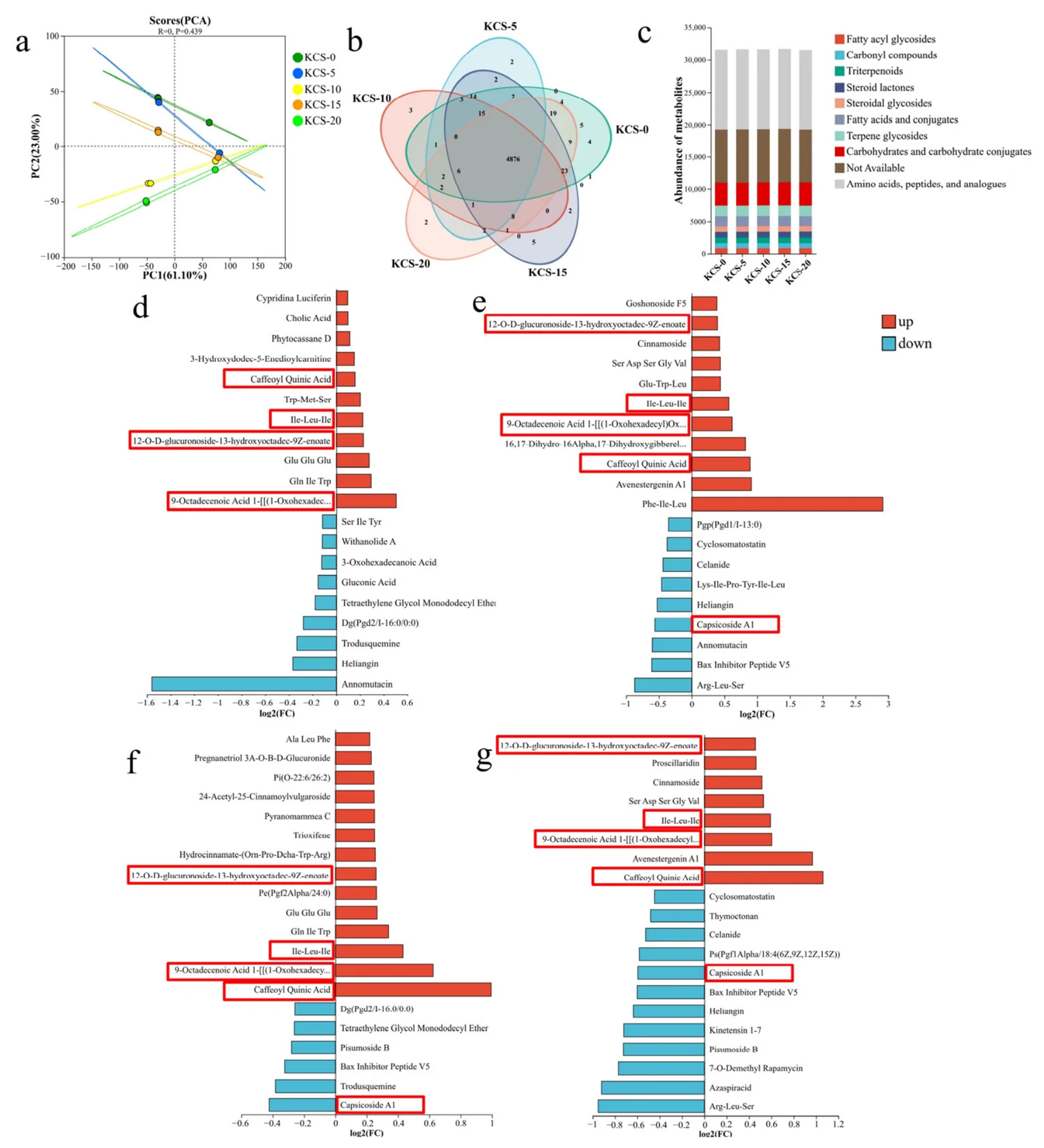
Metabolomic analysis of breads with different amounts of composite gel added. (**a**) PCA plot; (**b**) Venn diagram plot; (**c**) HMDB compound classification; (**d**–**g**) comparison of differential metabolites between KCS-0, KCS-5, KCS-10, KCS-15, and KCS-20. Red bars represent upregulation, blue bars represent downregulation, and the length of the bars represents the logarithm of the fold change. Bread with pure κ-carrageenan gel (KC); bread with bamboo shoot superfine powder (S); bread without any addition (KCS-0); breads with additions of 5%, 10%, 15%, and 20% of the bamboo shoot superfine powder composite gel (KCS-5, KCS-10, KCS-15, and KCS-20).

To further investigate the effects of different addition levels of the composite gel on bread flavor, untargeted metabolomic analysis was performed on five bread samples. LC-MS is one of the most widely used techniques for detecting non-volatile flavor substances [60]. The contributions of PC1 and PC2 were 61.10% and 23.00%, respectively, with a total contribution of 84.10%, indicating that the addition of the KCS had a dominant effect on the metabolome of bread. From the Venn diagram (Figure 9a,b), with increasing addition of KCS, the number of unique metabolites in each group showed an increasing trend, with KCS-20 having the highest number of unique metabolites.

Amino acids, as important non-volatile flavor components, are not volatile themselves but are important precursors for flavor formation. Under high temperatures, proteins undergo the Maillard reaction with reducing sugars, generating various flavor compounds such as pyrazines, furans, aldehydes, and ketones [61]. Figure 9c showed the distribution of major differential metabolite categories, including fatty acids and conjugates, carbohydrates and conjugates, amino acids, peptides and analogs, and terpene glycosides. From the differential metabolite comparison plots (Figure 9d–g), the upregulated peptides in the samples included small peptides, such as Trp-Met-Ser, Glu-Glu-Glu, Gln-Ile-Trp, and Ile-Leu-Ile. According to the basic component analysis of bamboo shoot shells, the protein content in bamboo shoot shells was 15.7% (Table S1). These small-molecule peptides may be derived from the decomposition of proteins in bamboo shoot superfine powders. During heating and baking, these small peptides can participate in the Maillard reaction to generate pyrazines, furans, and other compounds with baked, nutty, and caramel notes. Additionally, during Strecker degradation, small peptides release Strecker aldehydes (e.g., 3-methylbutanal from leucine, with a malty aroma), which are important components of the typical bread flavor [62,63]. Proteins can also affect flavor release by binding or adsorbing flavor compounds. Non-covalent bonds play a key role in the binding and adsorption between proteins and flavor compounds. For example, the molecular interactions between aldehyde carbonyl groups, whey or plant proteins, and carbonyl and alcohol flavor compounds not only lead to the migration of carbonyl groups from the interior to the edge of the flavor molecular structure but also enhance the binding efficiency between hexanal and proteins [64]. The increased content of small peptides helps bind more flavor compounds, resulting in a richer and more pronounced bread flavor.

In addition, four common upregulated metabolites were present across all four sample groups: Ile-Leu-Ile, caffeoyl quinic acid, 9-octadecenoic acid 1-[[(1-oxohexadecyl)oxy]methyl]-2-(phosphonoxy) ethyl ester, and 12-O-D-glucuronoside-13-hydroxyoctadec-9Z-enoate (Figure 9d–g). These compounds collectively contribute to the complex and multi-layered flavor characteristics of bread. Among them, Ile-Leu-Ile, a dipeptide containing branched-chain amino acids, enhances umami and kokumi, significantly improving the overall taste intensity, richness, and aftertaste duration of bread [65]. Caffeoyl quinic acid acts primarily as a bitterness modulator, partially suppressing or masking the undesirable bitter taste often found in whole-grain products while potentially promoting the formation of volatile aroma compounds [66]. When the addition level of KCS is between 10% and 20%, the content of Capsicoside A1 decreases (Figure 9e–g). Capsicoside A1 is a steroidal saponin, and the reduction in this compound may reduce the bitter taste of bread, thereby making the overall flavor more harmonious and purer.

In summary, the addition of KCS results in a fuller, richer, and more layered bread flavor, though attention should be paid to off-flavors resulting from lipid oxidation. At addition levels of 10–15%, the gel effectively regulates the release of small-molecule flavor compounds, improves the bread’s flavor, and enhances the overall sensory quality.

## 4. Conclusions

In this study, a bamboo shoot superfine powder/κ-carrageenan composite gel (KCS) was successfully constructed, and its application as a functional additive in bread was systematically investigated. This study demonstrated that the incorporation of bamboo shoot superfine powders into a κ-carrageenan system generated a denser and more continuous gel network through synergistic non-covalent interactions, primarily hydrogen bonding and electrostatic attraction. As a result, KCS exhibited enhanced viscoelasticity, freeze–thaw stability, and thermal stability compared with κ-carrageenan alone. When incorporated into bread, KCS markedly improved product quality, with the 10% addition level (KCS-10) showing the most pronounced effects. KCS-10 bread displayed improved color characteristics, reduced hardness and chewiness, and a more homogeneous gluten network structure. In addition, KCS suppressed the release of undesirable sulfur-containing volatiles while enriching favorable aroma compounds, leading to a more balanced aroma profile. Structural analyses further revealed that KCS reduced starch retrogradation and enhanced thermal stability, which contributed to improved texture retention during storage. Importantly, KCS-10 effectively delayed bread staling, as evidenced by a lower moisture loss and slower increases in hardness and chewiness over a 5-day storage period. Metabolomic profiling suggested that the upregulation of small peptides may promote flavor development through Maillard and Strecker reactions, providing a potential mechanistic basis for the observed sensory improvements. Collectively, these findings highlight the potential of bamboo shoot superfine powder/κ-carrageenan composite gels as a sustainable and effective strategy for enhancing bread quality, extending shelf lives, and valorizing bamboo shoot processing by-products.

## Data Availability

The data presented in this study are available on request from the corresponding authors due to privacy reason.

## Supplementary Materials

The following supporting information can be downloaded at: https://www.mdpi.com/article/10.3390/foods15183193/s1, Text S1: Metabolomics of bread with bamboo shoot superfine powder/κ-carrageenan composite gel; Table S1: The basic components of bamboo shoot shells; Table S2: Slope Analysis: Hardness vs Water loss rate.
