# Supplementary material for: Application of Bamboo Shoot Superfine Powder/κ-Carrageenan Composite Gel in Bread: Texture, Properties and Flavor
Source: Foods. 2026 Sep 9;15(18):3193. doi: 10.3390/foods15183193 (PMC13605932; doi:10.3390/foods15183193)
Supplement: Supplementary file 1 [file foods-15-03193-s001.zip › foods-4492183-supplementary.pdf]

Supplementary material

Text S1. Metabolomics of bread with bamboo shoot superfine powder/κ-carrageenan composite gel

Sample Preparation:Accurately transfer 200 μL of the sample into a 1.5 mL centrifuge tube, then add 800 μL of extraction solvent (methanol:acetonitrile= 1:1, v/v) containing four internal standards (e.g., L-2-chlorophenylalanine at 0.02 mg/mL). Vortex-mix for 30 s, followed by low-temperature ultrasonic extraction for 30 min (5℃, 40 kHz). The mixture is then placed at –20℃ for 30 min, centrifuged at 13,000g for 15 min (4℃), and the supernatant is transferred and evaporated to dryness under a nitrogen stream. The residue is reconstituted with 120 μL of reconstitution solution (acetonitrile:water=1:1, v/v), vortex-mixed for 30 s, and subjected to low-temperature ultrasonic extraction for 5 min (5℃, 40 kHz). After centrifugation at 13,000g for 10 min (4℃), the supernatant is transferred into an autosampler vial with an insert for LC-MS analysis. For quality control, an aliquot of 20 μL of supernatant from each sample is pooled to prepare a QC sample.

Chromatographic Conditions:Chromatographic separation is performed on an ACQUITY UPLC HSS T3 column (100 mm × 2.1 mm i.d., 1.8 μm; Waters, Milford, USA). Mobile phase A consists of 95% water and 5% acetonitrile (containing 0.1% formic acid), and mobile phase B consists of 47.5% acetonitrile, 47.5% isopropanol, and 5% water (containing 0.1% formic acid). The injection volume is 3 μL, and the column temperature is maintained at 40℃.

Mass Spectrometry Conditions:Electrospray ionization (ESI) is employed, and mass spectra are acquired in both positive and negative ion modes. The detection parameters are as follows: ion scan range of 70-1050 m/z; MS1 resolution of 60,000; MS2 resolution of 7,500; normalized collision energies of 20, 40, and 60. The raw data from MS analysis are processed using Compound Discoverer software for database searching, and finally the identification information of the sample components is obtained.

Table S1 The basic components of bamboo shoot shells

Basic composition	Content（g/100g）
Total Dietary Fiber	49.89
Cellulose	24.72
Hemicellulose	14.92
Lignin	23.03
Protein	15.7
Fat	0.8
Ash	4.68
Moisture	5.82

Table S2 Slope Analysis: Hardness vs Water loss rate.

Group	Slope	R^2
KCS-10	3322.0901	0.9680
KCS-5	3636.0625	0.9514
KCS-15	3682.6313	0.9220
KCS-0	3743.2452	0.9392
KC	3834.0641	0.9704
KCS-20	4060.7637	0.9762
S	5777.1230	0.968034
